# Ferroelectric-Antiferroelectric Transition of Hf_1–*x*_Zr_*x*_O_2_ on Indium Arsenide with Enhanced Ferroelectric Characteristics for Hf_0.2_Zr_0.8_O_2_

**DOI:** 10.1021/acsaelm.2c01483

**Published:** 2022-12-14

**Authors:** Hannes Dahlberg, Anton E. O. Persson, Robin Athle, Lars-Erik Wernersson

**Affiliations:** † Department of Electrical and Information Technology, Division of Electromagnetics and Nanoelectronics, Lund University, Box 118, 221 00Lund, Sweden; △ NanoLund, Lund University, Box 118, 221 00Lund, Sweden

**Keywords:** ferroelectric, antiferroelectric, III-V, polarization, hysteresis, endurance, permittivity, coercive field

## Abstract

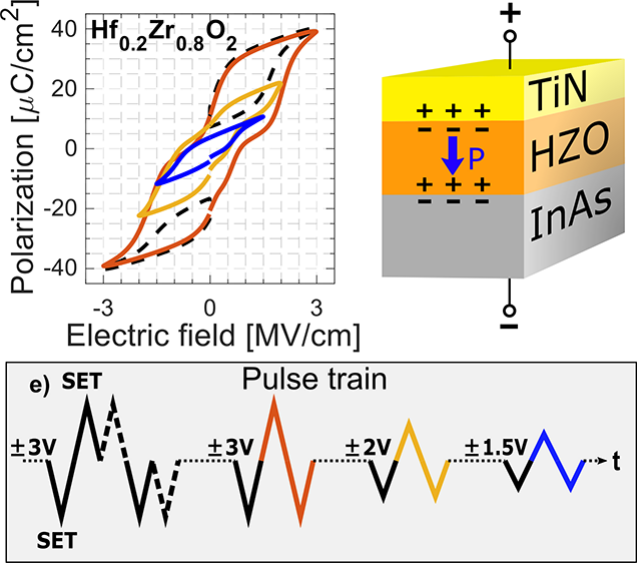

The ferroelectric (FE)–antiferroelectric (AFE) transition in Hf_1–*x*_Zr_*x*_O_2_ (HZO) is for the first time shown in a metal–ferroelectric–semiconductor (MFS) stack based on the III-V material InAs. As InAs displays excellent electron mobility and a narrow band gap, the integration of ferroelectric thin films for nonvolatile operations is highly relevant for future electronic devices and motivates further research on ferroelectric integration. When increasing the Zr fraction *x* from 0.5 to 1, the stack permittivity increases as expected, and the transition from FE to AFE-like behavior is observed by polarization and current–voltage characteristics. At *x* = 0.8 the polarization of the InAs-based stacks shows a larger FE-contribution as a more open hysteresis compared to both literature and reference metal–ferroelectric–metal (MFM) devices. By field-cycling the devices, the switching domains are studied as a function of the cycle number, showing that the difference in FE–AFE contributions for MFM and MFS devices is stable over switching and not an effect of wake-up. The FE contribution of the switching can be accessed by successively lowering the bias voltage in a proposed pulse train. The possibility of enhanced nonvolatility in Zr-rich HZO is relevant for device stacks that would benefit from an increase in permittivity and a lower operating voltage. Additionally, an interfacial layer (IL) is introduced between the HZO film and the InAs substrate. The interfacial quality is investigated as frequency-dependent capacitive dispersion, showing little change for varying ZrO_2_ concentrations and with or without included IL. This suggests material processing that is independently limiting the interfacial quality. Improved endurance of the InAs-Hf_1–*x*_Zr_*x*_O_2_ devices with *x* = 0.8 was also observed compared to *x* = 0.5, with further improvement with the additional IL for Zr-rich HZO on InAs.

## Introduction

The ferroelectric behavior in hafnium oxide (HfO_2_) has been intensely studied since its discovery in 2011.^1^ Ferroelectric thin films have gathered immense popularity in implementations such as ferroelectric random-access memory (FeRAM), ferroelectric tunnel junctions (FTJs), and ferroelectric field-effect transistors (FeFETs), which are regarded as promising candidates for nonvolatile memory cells and neuromorphic computing due to their ultralow power consumption and fast operations.^2−5^ HfO_2_ has the additional benefit of being highly compatible in current CMOS processes and back-end-of-line (BEOL) architectures, making further research of ferroelectric HfO_2_ highly motivated.^6,7^ A solid mixture of HfO_2_ and zirconium oxide (ZrO_2_) as Hf_1–*x*_Zr_*x*_O_2_ is presently the standard in ferroelectric device research, commonly with *x* = 0.5.^8,9^ By varying *x*, different crystal phases can be favored, where an *x* near 0.5 is shown to favor the ferroelectric orthorhombic phase the most.^9,10^ A HfO_2_ thin film crystal consists mainly of monoclinic phase without any ferroelectric behavior, while an increase of the Zr concentration from 50 to 100% eventually leads to a transition from orthorhombic to tetragonal phase with volatile anti-ferroelectric (AFE) behavior and a field-driven transition to FE orthorhombic phase;^7,8^ thus, the transition between FE to AFE thin films show a mixture of these characteristics.

While silicon is the basis of modern CMOS technology, the material is approaching its inherent scaling and operating frequency limit.^11^ III-V semiconductors, such as indium-arsenide (InAs), provide advantageous electronic properties such as ultrahigh electron mobility, narrow band gap, and high-frequency response relevant for low-power and high-speed devices but also of interest for low-power FTJs and FeFETs.^12−14^

Ferroelectric HfO_2_ is extensively characterized in standard device structures such as MFM, MFS, and metal–ferroelectric–insulator–semiconductor (MFIS). There are several reports in the literature describing the properties of HZO films at various ZrO_2_ concentrations in classic MFM structures including the now well-established FE-AFE transition.^8,9,15,16^ By increasing the ZrO_2_ concentration, the permittivity of the film is increased, and the thermal processing temperature required is expected to decrease as ZrO_2_ has a lower crystallization and transition temperature than HfO_2_.^8,17,18^ Another promising property for AFE-based memories are relatively low coercive fields E_C_, enabling lower operating voltages, leading to reduced power consumption and electrical stress, as well as better endurance compared to purely ferroelectric HZO.^19−21^ In this work, we study HZO-based ferroelectric MFS and MFIS capacitors on InAs with varying ZrO_2_ concentrations, where we focus on the FE-AFE transition and the electrical properties, comparing it with that of standard MFM structures. An interfacial layer (IL) of approximately 1.2 nm-thin Al_2_O_3_ between the HZO and InAs is included as an insulator in the MFIS structure and compared alongside the MFS devices. The switching currents, polarization hysteresis, and capacitance–voltage dependencies are analyzed and compared. The InAs-based Hf_0.2_Zr_0.8_O_2_ MFS and MFIS devices show more ferroelectric-like nonvolatile switching compared to identical MFM devices with expected AFE-like behavior, enabling the use of Zr-rich HZO thin films as nonvolatile ferroelectric elements.

## Experimental Section

MFS devices are fabricated as a top electrode (TE)-HZO stack directly deposited on low-doped InAs (100) substrate (n-type, 1.7e16 cm^–3^), acting as a bottom electrode (BE). After the removal of the native InAs surface oxide by HCl etching, the insulating materials are immediately deposited by atomic layer deposition (ALD) in a Picosun thermal ALD reactor. In the case when an Al_2_O_3_ IL of ∼1.2 nm (12 cycles) is included, it is first deposited with the precursor TMAl cycled with H_2_O as purging gas. The TMAl source was kept at room temperature. The Al_2_O_3_ deposition is followed by deposition of ∼10 nm (100 cycles) HZO with varying Zr concentrations using conventional precursors TDMA(Hf) and TEMA(Zr), also here using H_2_O as purging gas/the oxygen source. The TDMA(Hf) and TEMA(Zr) sources are heated to 100 and 110 °C, respectively. All ALD films were deposited at a chamber temperature of 200 °C. To vary the Zr concentration in the 10 nm-thick HZO film, the material is deposited with a pulsing scheme according to Table 1.

**Table 1:** ALD Pulse Scheme of Hf–Zr for Different *x* Values in Hf_1–*x*_Zr_*x*_O_2_ Compositions

*x*	Hf pulses	Zr pulses	no. of repetitions
0.5	1	1	50
0.66	1	2	33^a^
0.8	1	4	20
1	0	1	100

a Plus one additional Hf pulse.

After ALD deposition, a 10 nm-thick TE of TiN is deposited using physical vapor deposition (PVD) in an AJA Orion sputter tool. A post-deposition rapid thermal annealing is then performed for all devices at 550 °C for 30 s. The individual devices are defined by UV lithography as circular planar capacitors with a radius ranging from 8 to 50 μm. A contacting layer of Ti (5 nm) and Au (200 nm) is deposited on the TE by electron-beam evaporation in a Temescal E-Beam Evaporation tool. After a lift-off process, the exposed TiN between devices is removed in a wet-etch process using a heated ammonia-peroxide mix. An MFM reference sample is identically processed except for the BE being PVD-deposited TiN (with identical parameters as for the TE) on Si without initial native oxide etching.

The devices are electrically characterized in a TS2000-SE semi-automatic probe station using a Keysight B1500 parameter analyzer equipped with B1530A waveform generators. The virtual ground measurement scheme is used for measuring ferroelectric switching currents with triangular voltage pulses of alternating positive and negative amplitude, consequently measuring the displacement polarization versus applied electric field.^22^ The devices were (unless otherwise specified) subject to a wake-up sequence of 1000 square pulses (1 kHz) prior to measurements at a corresponding voltage. To measure the ferroelectric response separately from current leakage in devices at large biases, measurements were done with 1 and 10 kHz pulses with current amplitude normalization corresponding to 1 kHz voltage pulses. The capacitance measurements were performed with an Agilent 4294A impedance analyzer. A DC bias is swept between a higher and lower limit, with an added AC voltage bias of 50 mV at a certain frequency. Before each C–V measurement, the device was subject to a 5-cycle voltage sweep (±2.5 V) as a form of wake-up. Additionally, grazing incidence X-ray diffraction (GIXRD) measurements were performed using a Bruker D8 Diffractometer with a Cu Kα source and a 0.5^○^ incidence angle.

## Results & Discussion

**Figure 1:**
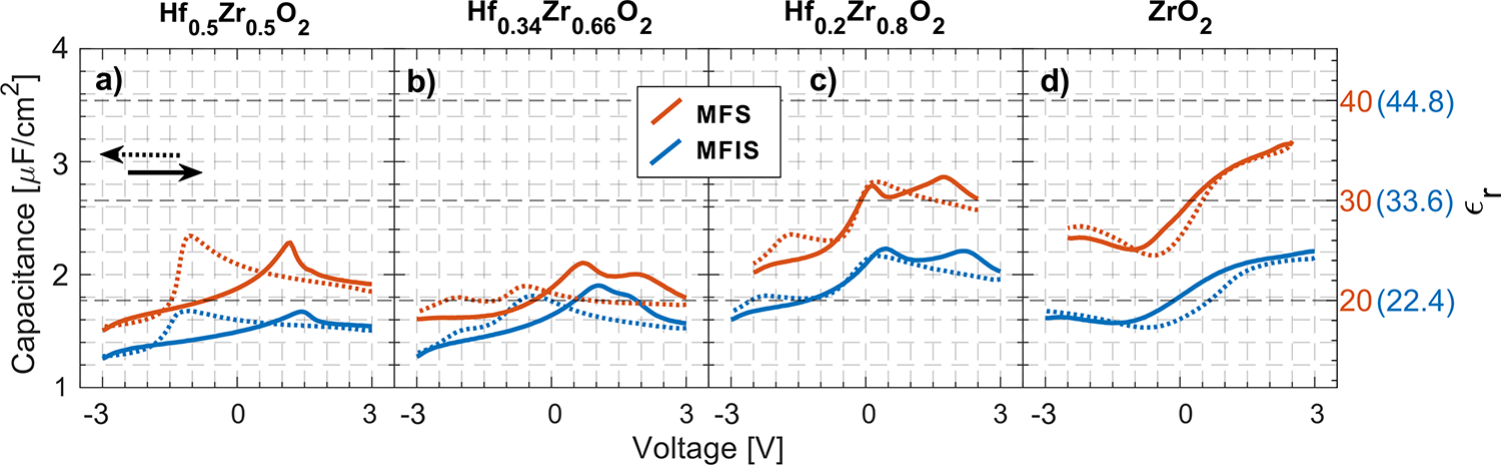
CV characteristics measured at 1 MHz 50 mV AC bias for 10 nm HZO (and 1.2 nm Al_2_O_3_ IL) for MFS and MFIS capacitors with different ZrO_2_ concentrations: (a) 50, (b) 66, (c) 80, and (d) 100%. Bias sweep direction is indicated by the arrows. The left-hand axis corresponds to the capacitance, the right-hand axis to the insulator stack ε_r_ (only considering the dielectric thickness of MFS (orange) and MFIS (blue)).

For initial investigation into the effect of both varying *x* in the Hf_1–*x*_Zr_*x*_O_2_ film and inclusion of an IL in InAs MFS and MFIS devices, the capacitance–voltage dependence is studied in Figure 1. The inclusion of an Al_2_O_3_ IL would decrease the overall capacitance, according to the series summation of capacitors. For an increasing ZrO_2_ concentration, the capacitance increases as expected, with Hf_0.5_Zr_0.5_O_2_ to Hf_0.2_Zr_0.8_O_2_ (Figure 1a–c) displaying conventional FE and mixed-FE-AFE switching peaks in the CV diagram.^8^ The Zr devices (Figure 1d) show very little sign of AFE switching, discussed later in the *P*–*V* and *I*–*V* analyses.

The extracted permittivity in the right-hand axis in Figure 1 is only considering a dielectric film between two electrodes with a thickness of 10 nm (11.2 nm with an IL included) and not the capacitive contribution from the semiconductor inversion layer,^23^ but it nevertheless gives an idea of the trend. The 80% Zr sample shows the peak permittivity at biases close to zero both with and without included IL while also displaying FE-AFE switching when comparing the voltage sweeps from different directions. This composition is of interest and is further electrically investigated below.

To evaluate the interfacial material quality, we vary the frequency of the AC component, and a relative change in frequency dispersion can be observed as the relative decrease in capacitance when increasing the AC bias frequency. The time constant of trapped charges is assumed to be larger than that of the majority carrier response due to tunneling distances into or out of the oxide and differences in activation energy resulting in a lower capacitance for higher frequency AC signals.^24^ By studying the relative change Δ*C* for each frequency decade, the defect concentration in the FE–semiconductor interface can be relatively compared between different samples.^25,26^

Figure S1 in the Supporting Information shows the capacitance–voltage response when increasing the AC bias frequency from 10 kHz to 1 MHz for MFS and MFIS devices with varying ZrO_2_ concentration. In Figure S2, the change in capacitance at 2 V bias per frequency decade is illustrated for voltage sweeps from negative to positive bias. The dispersion is similar for all ZrO_2_ concentrations independently of an IL, ranging between 5.3–6.2 and 5.8–6.4% with and without an IL, respectively, which is comparable to previously reported values on InAs/HZO.^27^ This indicates that the semiconductor–oxide interface quality regarding interface states and border traps is relatively similar for MFS and MFIS devices and independent of the ZrO_2_ concentration. This also suggests an overall InAs–oxide interface quality limiting processing step such as thermal annealing or preoxide deposition treatment or the oxide deposition itself.

A consequence of including an IL is the change in voltage drop over the HZO layer. The total capacitance decreases due to a larger total insulator thickness, and the effective voltage division with respect to oxide thickness and relative permittivity is given as
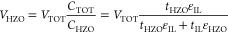
1

For the case where *t*_HZO_ = 10 nm and *t*_IL_ = 1.2 nm and using ε_r HZO_ = 25, ε_r IL_ = 9,^28^ the voltage over the HZO film is 75% of the total bias. Either an increased relative permittivity or a decreased thickness of the IL results in a voltage drop over the HZO approaching the applied voltage. The thickness of the IL is thus a critical parameter when considering bias, as illustrated by eq 1. This is important to consider when comparing polarization–voltage responses of samples with different thicknesses.

**Figure 2:**
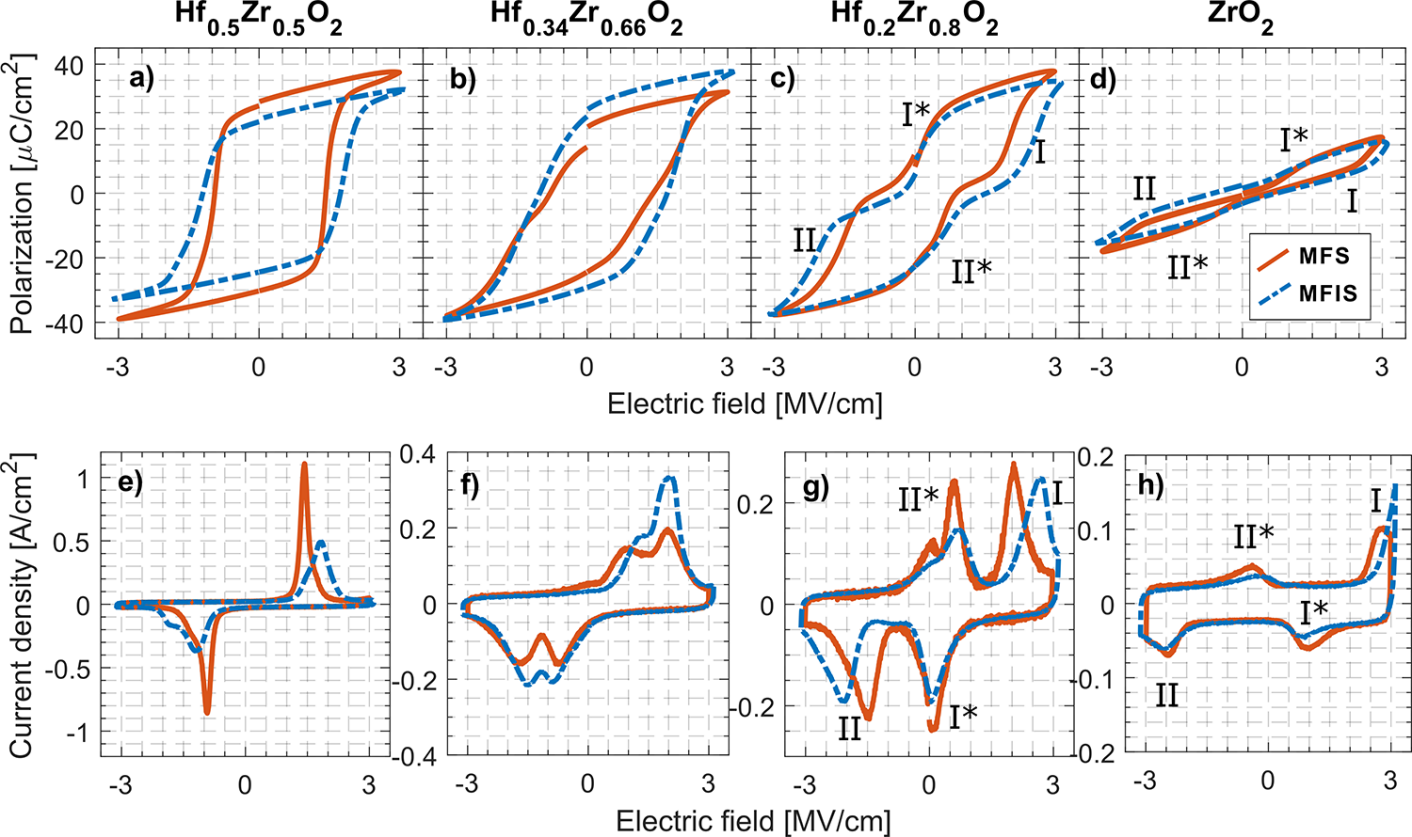
Polarization–voltage hysteresis and current–voltage response for different ZrO_2_ concentrations: (a, e) 50, (b, f) 66, (c, g) 80, and (d, h) 100% in MFS (metal–ferroelectric–semiconductor) and MFIS (metal–ferroelectric–insulator–semiconductor) configurations. All currents are normalized to the device area. For 80 and 100% ZrO_2_, observed switching peaks and their corresponding hysteresis flanks are denoted as **I**, **II**, I*, II*.

Figure 2 shows the measured P-E hysteresis curves and switching currents of the devices for varying Hf–Zr concentration ratios, with and without an included Al_2_O_3_ IL. For Hf_0.2_Zr_0.8_O_2_ and ZrO_2_, **I** and I* (**II** and II*) refer to the observed switching current pairs and their corresponding hysteresis flanks. The IL samples were subjected to a larger bias (after identical wakeup as for non-IL samples), so the HZO voltage drop were comparable to non-IL samples. As a result of the asymmetric BE and TE, the oxide stack is exposed to a built-in electrical field. This built-in bias results in a shift of the coercive field *E*_C_ corresponding to the flat band voltage *V*_FB_ (corresponding to the bias required to make the semiconductor energy bands flat toward the dielectric), effectively shifting the polarization hysteresis midpoint to *V*_FB_. The semiconductor depletion layer will contribute to the change in the applied bias due to an increased barrier thickness in depletion, which contributes to asymmetric current switching between positive and negative biases.^29^

The switching currents for Hf_0.34_Zr_0.66_O_2_ and Hf_0.2_Zr_0.8_O_2_ in Figure 2f,g display a current peak splitting for the positive switching current (II* in Figure 2g). This is observed in other works^30,31^ and is attributed to internal bias fields created by defect-related charge injection of oxygen vacancies. By observing this split for positive biases, we suspect the bottom HZO-InAs interface as the major site of the defects,^30^ in line with previous XPS measurements.^32^

In Figure 2a,e the typical FE hysteresis is observed for Hf_0.5_Zr_0.5_O_2_. The IL sample yields a smaller remanent polarization *P*_r_ compared to the non-IL sample at a fixed bias as a larger bias is required for complete switching. The switching currents have both been separated out toward higher bias fields due to the HZO-IL capacitive voltage divider. For Hf_0.34_Zr_0.66_O_2_ (Figure 2b,f), the increase of t-phase ZrO_2_ creates a larger distribution of switching fields around *E*_C_, which results in less steep switching flanks.^22^ The switching currents for both the IL and non-IL samples show two separate switching peaks, indicating domains with differently distributed internal bias fields. For the ZrO_2_ film (Figure 2d,h), the non-IL MFS sample is measured at 3 V bias amplitude with wake-up pulses of 2.5 V, while the IL MFIS sample is measured at 3.5 V bias amplitude with wake-up pulses of 3 V. The hysteresis is thinner and pinched at zero bias for both samples, resembling a weak but conventional antiferroelectric hysteresis with **I** and I* (**II** and II*) located on the same side of the zero bias.^8,22^ As the electrical fields needed for AFE tetragonal-orthorhombic transition and switching are expected to be higher for larger ZrO_2_ concentrations,^10^ the positive bias needed to reach the **I** domain for the IL sample is larger than the one reached before the leakage current starts to dominate. This is observed more clearly when including an IL in Figure 2h due to the lower actual electrical field over the HZO layer and might be a reason for not clearly observing the switching characteristics in the capacitive measurements above.

For Hf_0.2_Zr_0.8_O_2_ (Figure 2c,g), the polarization shape is shifted toward a pinched hysteresis due to a more separated switching of I, II* and **II**, I* over the applied electrical field. When **I** and I* (**II** and II*) are located on the same sides of the zero bias, back-switching occurs and gives the hysteresis the characteristically pinched AFE hysteresis.^22^ In Figure 2g, II* is mainly located in the same quadrant as **I**, while I* is located close to zero bias. Because of **I** and I* (**II** and II*) not being located entirely on the same side of the zero bias, the resulting switching characteristic is a hysteresis not completely pinched at zero bias, still retaining some degree of polarization at zero bias as can be observed in Figure 2c. For both the IL (MFIS) and non-IL (MFS) samples, the existence of switching domains with different coercive fields is apparent.^22^ The IL sample shows less back-switching from I* and II* around zero bias while being comparable to the non-IL sample at larger biasing. To note is also the shift in switching fields when including the IL for **I** (2.0 to 2.6 MV/cm) and **II** (−1.5 to −2.1 MV/cm), which is to expect from the capacitive voltage division, while I* and II* are located at identical bias fields in both cases.

As discussed above, the reports in the literature of FE-AFE transitions by varying the Zr concentration in HZO mainly cover conventional TiN-based MFM stacks. Stacks with ZrO_2_ > 60% generally show more AFE-like behavior than observed at a ZrO_2_ concentration of 80% in this study.^8,9,15,16^ Our Hf_0.2_Zr_0.8_O_2_ sample is therefore of interest as it exhibits the highest peak permittivity of the studied HZO compositions as well as a relatively open hysteresis (some degree of nonvolatile polarization) compared to previously studied conventional stacks of similar composition. Although the *P*_r_ is low for Hf_0.2_Zr_0.8_O_2_ compared to Hf_0.5_Zr_0.5_O_2_, it is still relevant for use in devices such as FeFETs where the polarization switching is not used for generating a detectable switching current but for modulating the electrostatic potential.^33^ For further analysis, an MFM reference sample is produced side by side with an InAs MFS sample with identically deposited 10 nm Hf_0.2_Zr_0.8_O_2_ films.

**Figure 3:**
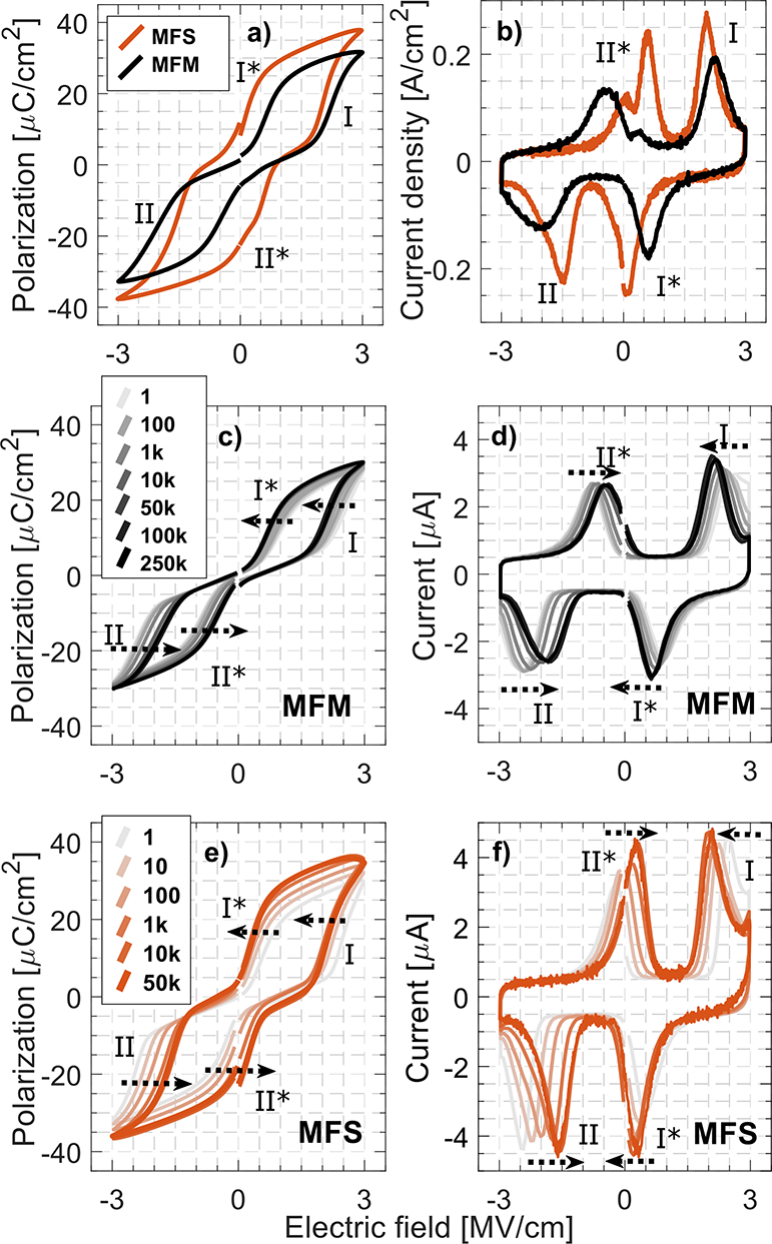
Polarization–voltage and current–voltage comparisons of MFM (black) and InAs MFS (orange) Hf_0.2_Zr_0.8_O_2_ devices. (a, b) *P*–*V* and *I*–*V* characteristics after identical wakeup for both devices. (c–f) Field cycling of pristine devices with identical area, with arrows indicating the movement of *E*_C_ for the switching current pairs and hysteresis flanks **I**, I* (**II**, II*).

The structural properties and the crystal composition of the Hf_0.2_Zr_0.8_O_2_ MFM and MFS (without Al_2_O_3_) samples are investigated by GIXRD. Figure S3 in the Supporting Information shows the diffraction spectrum where peaks of intensity can be found at identical angles for both MFM and MFS samples, confirming a t(011)/o(111) crystal phase composition.^8^ This peak is comparable to previously reported GIXRD measurements on InAs-based Hf_0.5_Zr_0.5_O_2_ MFS capacitors.^34^ Thus, electrical measurements of the two samples are employed to further study the difference in nonvolatile behavior. Identical pulsed measurements are compared in Figure 3a,b, where a clear difference in domain switching bias is observed. The switching current peaks (Figure 3b) **I** and II* are for the MFS located mostly in the same quadrant, while for the MFM, they are predominantly separated on either side of the zero bias. Similarly, I* is located partially in the same quadrant as the **II** switching domain, which is not the case for the MFM sample where I* and **II** are separated by the zero bias. This is seen in the *P*–*V* curve as a widening of the hysteresis at I* and II* for the MFS sample.

The inherent differences between MF(*I*)S stacks compared to a TiN-based MFM stack need to be considered when evaluating the presented results. Depolarization fields due to different charge screening lengths in the metal BE and semiconductor BE as well as “dead layer” effects could play a role in the migration of the switching domains I* and II*.^35^ The depolarization field over an interfacial layer can be expressed as^35^
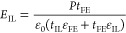
2where *P* is the ferroelectric polarization surface charge. As the field over any interfacial layer (“dead” or Al_2_O_3_) in series with the HZO layer can become very large if *t*_FE_ > *t*_IL_ and ε_FE_ > ε_IL_, injection of charge carriers can occur between the interfacial layer and HZO and could then be a cause of imprint and switching domain voltage shifts.^36,37^ Further, considering that a lower HZO crystallization temperature for ferroelectric InAs MFS stacks compared to TiN-based MFM stacks has been reported could also indicate that the InAs-HZO interface plays a role in FE domain stabilization.^34^

Pristine Hf_0.2_Zr_0.8_O_2_ samples were additionally field cycled with a 10 kHz triangular wave to investigate the movements of the switching domains in the bias field and to rule out any unseen wakeup behavior and domain depinning.^38,39^Figure 3c–f shows the field cycling of both the MFM and MFS device. The MFS sample is cycled 50,000 times, while the MFM sample is cycled up to 250,000 times. As indicated by the black arrows, the switching domains move toward a lower bias threshold both for the MFM and MFS samples. While the MFM sample was put through 2,000,000 more cycles than the MFS sample, the MFM sample displays less tendencies of migrating the I* and II* across the zero bias, still retaining its AFE-like characteristic after field cycling.

**Figure 4:**
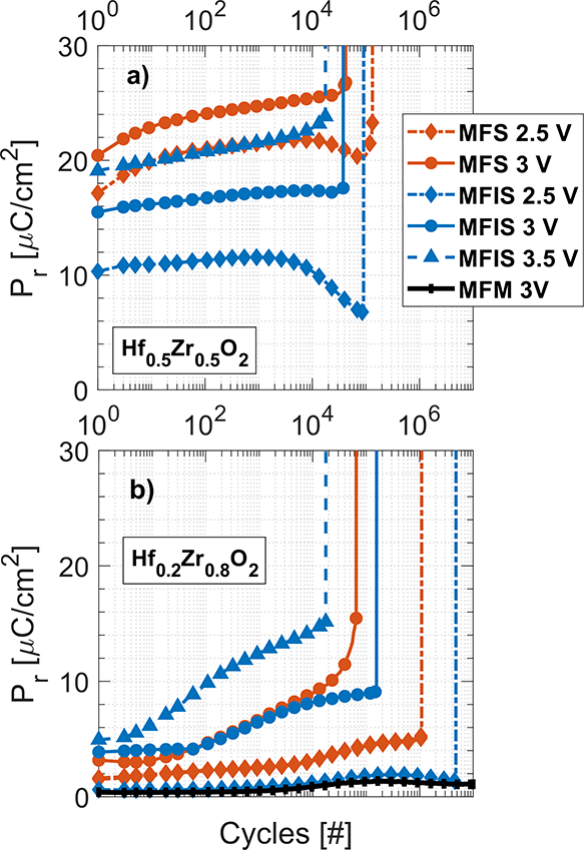
Remanent polarization *P*_r_ endurance as a function of field cycling for different pulse amplitudes for (a) Hf_0.5_Zr_0.5_O_2_ and (b) Hf_0.2_Zr_0.8_O_2_. The cycling and measurements are performed with 10 kHz triangular pulses. Here, *P*_r_ represents the total polarization window 2*P*_r_ at zero bias divided by two.

The endurance properties of the devices were also evaluated and compared. The *P*_r_ (defined as half of the total remanent polarization window 2*P*_r_ at zero bias) as a function of field cycling is shown in Figure 4a,b for pristine devices with 50 and 80% ZrO_2_ compositions, respectively. For Hf_0.5_Zr_0.5_O_2_, the MFS and MFIS devices sustain a similar number of cycles before experiencing breakdown at respective electric fields, with no increase in the cycle number for MFIS. However, Figure 4b shows an increase in endurance for the Hf_0.2_Zr_0.8_O_2_ devices, most notable at lower electric fields. For Hf_0.2_Zr_0.8_O_2_, the endurance benefit from the introduction of an interfacial layer is also visible, as the MFIS samples for all equal biasing experiences higher cycle numbers before breakdown. Additionally, a gradual breakdown can be seen as the nonsharp transition to the vertical line in Figure 4, (mainly for the MFS devices and most prominently for Hf_0.2_Zr_0.8_O_2_ at 3 V) due to a gradual increase in leakage current. Notably, Figure 4b shows a substantial increase in *P*_r_ with the number of cycles until breakdown for both Hf_0.2_Zr_0.8_O_2_ MFS and MFIS samples at 3 and 3.5 V. The MFM and MFIS (at 3 and 2.5 V, respectively) devices show both a small increase in remanent polarization and fatigue at the end of the cycling.

An increase from 50 to 80% ZrO_2_ in HZO on InAs thus seems to increase the endurance properties with respect to the number of cycles but with an observed gradual increase of leakage current before breakdown. The introduction of an interfacial layer is thus more beneficial for Hf_0.2_Zr_0.8_O_2_ than for Hf_0.5_Zr_0.5_O_2_, increasing the endurance further by decreasing the detrimental leakage.

**Figure 5:**
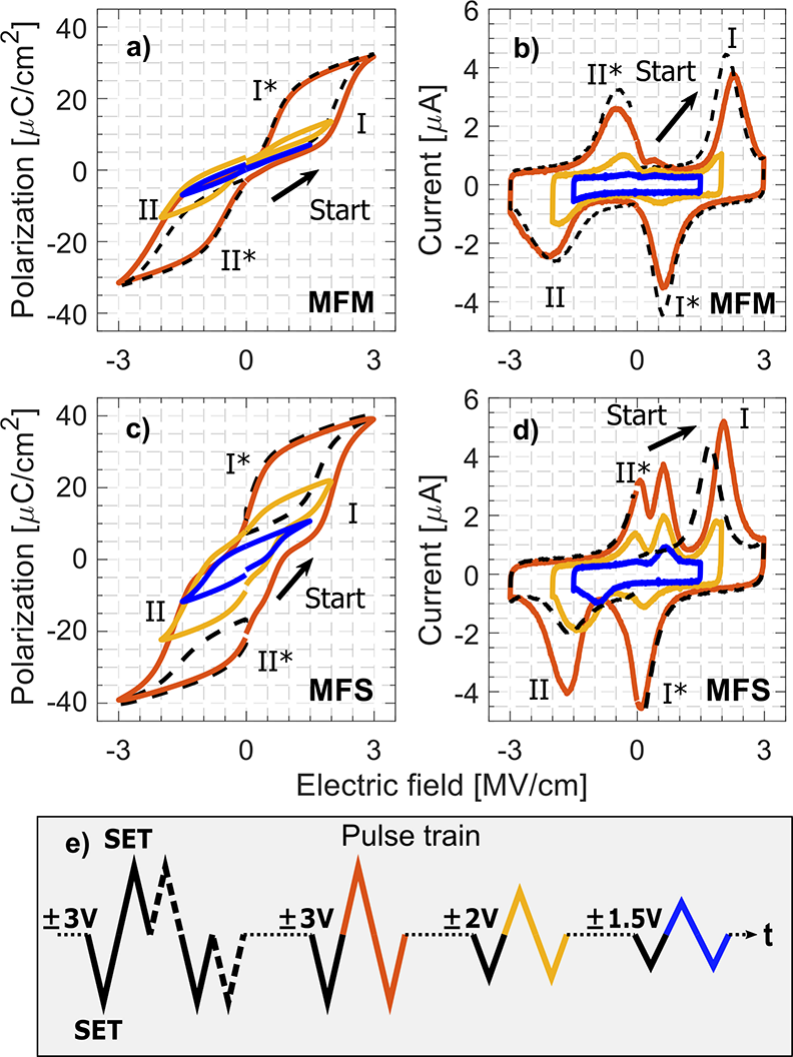
Polarization–voltage and current–voltage characteristics of (a, b) MFM and (c, d) MFS Hf_0.2_Zr_0.8_O_2_ devices. (e) Applied 10 kHz pulse train of varying amplitude, where the color and line type correspond to the resulting hysteresis and currents in (a–d). First, identically consecutive pulses yield the volatile AFE part of the switching process (dashed black line), before opening the hysteresis at maximum bias (±3 V). Lowering the bias in steps finally yields the nonvolatile switching contribution. All measured pulses are initialized by a “SET” pulse (solid black line).

To illustrate the AFE-like back-switching and to further separate the FE-like nonvolatile contribution to the overall switching, a pulse train as illustrated in Figure 5e is applied to MFM and MFS Hf_0.2_Zr_0.8_O_2_ devices (see Figure S4 in the Supporting Information for MFIS). The measured pulses are initialized with a “SET” pulse to ensure a controlled precondition. By following a SET pulse with a measured pulse of identical polarity and amplitude, the amount of volatility in the switching can be observed as the overlap of the dashed black lines to the solid lines in Figure 5. The MFM sample in Figure 5a,b displays near-identical *P*–*V* and *I*–*V* characteristics for completely switching ±3 V pulses and consecutive matching-polarity pulses indicating AFE behavior and volatile switching, with the lower ±1.5 V bias displaying an almost purely dielectric behavior. However, for the MFS sample in Figure 5c,d, there is a significant difference between the *P*–*V* and *I*–*V* curves of complete switching at ±3 V bias and the consecutive matching-polarity pulses, where AFE-typical back-switching is decreased. This is most noticed for negative biases, at **II** and II*. The small negative-bias back-switching current at II* for ±2 V is removed when lowering the pulse bias to ±1.5 V where it then is possible to isolate the switching to an FE-like degree. No back-switching from **I** at I* takes place since **I** is not reached at low biasing due to the asymmetric switching in the MFS stack. This minor-loop hysteresis shown as the blue curve in Figure 5c illustrates the accessible FE nonvolatile switching and the relatively low voltages needed to access it. As discussed above, a low *P*_r_ is not necessarily detrimental to memory operations where the polarization is used for electrostatic control. However, the reduction of the coercive field *E*_C_, clearly seen in the ±1.5 V subloop in Figure 5c, does reduce the maximum memory window in FeFETs.^40^ In such cases, the tradeoff for a lower memory window is the lower operating voltages, which would mitigate unwanted effects such as stress-induced defect generation.^20^

## Conclusions

In this work, we have investigated the effects on ferroelectric polarization by varying *x* in Hf_1–*x*_Zr_*x*_O_2_ thin film TiN-HZO-InAs metal-oxide-semiconductor capacitors. Additionally, an interfacial layer of 1.2 nm Al_2_O_3_ was in a parallel sample series included between the ferroelectric film and the semiconductor for comparison. Capacitance measurements confirm a trend of increasing permittivity in the stack as the Zr concentration increased. As expected, when varying *x* in Hf_1–*x*_Zr_*x*_O_2_ from 0.5 to 1, we observe an FE-AFE transition as an observable shift in *C*–*V* and *P*–*V* characteristics. At high Zr concentrations (80%), the InAs-based capacitors show a larger component of nonvolatile switching compared to reported values and reference MFM capacitors. A nonvolatile polarization hysteresis is measured when decreasing the bias in a switching pulse train, corresponding to the ferroelectric contribution in the total switching. Our data show that ferroelectric devices with a higher Zr concentration in the stack exhibit a higher relative permittivity and lower operating voltage for nonvolatile behavior, although the remanent polarization is somewhat reduced. The inclusion of an IL does not alter the polarization characteristics in a substantial way, but as the voltage drop over the HZO decreases due to a capacitive voltage division, the domain switching fields increase. The polarization endurance however shows a clear benefit of an included IL for Hf_0.2_Zr_0.8_O_2_ devices compared to Hf_0.5_Zr_0.5_O_2_, with an improved endurance for the 80% ZrO_2_ HZO composition. The inclusion of an IL is of interest for functional integration and interface engineering and might prove to be the key in achieving high-performance FE-based devices integrated on III-V semiconductors.

## Supporting Information Available

Measurements of capacitive dispersion, GIXRD measurements of Hf_0.2_Zr_0.8_O, and additional electrical characterization of MFIS (PDF)

## Notes

The data that support the findings of this study are available from the corresponding author upon reasonable request.

## References

[ref1] Böscke T. S.; Müller J.; Bräuhaus D.; Schröder U.; Böttger U. Ferroelectricity in Hafnium Oxide Thin Films. Appl. Phys. Lett. 2011, 99, 102903 10.1063/1.3634052.

[ref2] Marković D.; Mizrahi A.; Querlioz D.; Grollier J. Physics for Neuromorphic Computing. Nat. Rev. Phys. 2020, 2, 499–510. 10.1038/s42254-020-0208-2.

[ref3] Chen A. A Review of Emerging Non-Volatile Memory (NVM) Technologies and Applications. Solid-State Electron. 2016, 125, 25–38. 10.1016/j.sse.2016.07.006.

[ref4] Park M. H.; Lee Y. H.; Mikolajick T.; Schroeder U.; Hwang C. S. Review and Perspective on Ferroelectric HfO2-Based Thin Films for Memory Applications. MRS Commun. 2018, 8, 795–808. 10.1557/mrc.2018.175.

[ref5] Majumdar S. Back-End CMOS Compatible and Flexible Ferroelectric Memories for Neuromorphic Computing and Adaptive Sensing. Adv. Intel. Syst. 2022, 2100175 10.1002/aisy.202100175.

[ref6] Mulaosmanovic H.; Breyer E. T.; Dünkel S.; Beyer S.; Mikolajick T.; Slesazeck S. Ferroelectric Field-Effect Transistors Based on HfO2: A Review. Nanotechnology 2021, 32, 502002 10.1088/1361-6528/ac189f. 34320479

[ref7] Schroeder U.; Park M. H.; Mikolajick T.; Hwang C. S. The Fundamentals and Applications of Ferroelectric HfO2. Nat. Rev. Mater. 2022, 653 10.1038/s41578-022-00431-2.

[ref8] Müller J.; Böscke T. S.; Schröder U.; Mueller S.; Bräuhaus D.; Böttger U.; Frey L.; Mikolajick T. Ferroelectricity in Simple Binary ZrO 2 and HfO 2. Nano Lett. 2012, 12, 4318–4323. 10.1021/nl302049k. 22812909

[ref9] Park M. H.; Lee Y. H.; Kim H. J.; Kim Y. J.; Moon T.; Kim K. D.; Muller J.; Kersch A.; Schroeder U.; Mikolajick T.; Hwang C. S. Ferroelectricity and Antiferroelectricity of Doped Thin HfO 2 -Based Films. Adv. Mater. 2015, 27, 1811–1831. 10.1002/adma.201404531. 25677113

[ref10] Park M. H.; Kim H. J.; Kim Y. J.; Lee Y. H.; Moon T.; Kim K.; Hyun S. D.; Fengler F.; Schroeder U.; Hwang C. S. Effect of Zr Content on the Wake-Up Effect in Hf 1– xZrxO2 Films. ACS Appl. Mater. Interfaces 2016, 8, 15466–15475. 10.1021/acsami.6b03586. 27237137

[ref11] Theis T. N.; Solomon P. M. In Quest of the “Next Switch:” Prospects for Greatly Reduced Power Dissipation in a Successor to the Silicon Field-Effect Transistor. Proc. IEEE 2010, 98, 2005–2014. 10.1109/JPROC.2010.2066531.

[ref12] Persson A. E. O.; Zhu Z.; Athle R.; Wernersson L.-E. Integration of Ferroelectric HfxZr1-XO2 on Vertical III-V Nanowire Gate-All-around FETs on Silicon. IEEE Electron Device Lett. 2022, 1–1. 10.1109/LED.2022.3171597.

[ref13] del Alamo J. A. Nanometre-Scale Electronics with III–V Compound Semiconductors. Nature 2011, 479, 317–323. 10.1038/nature10677. 22094691

[ref14] Kilpi O.-P.; Hellenbrand M.; Svensson J.; Persson A. R.; Wallenberg R.; Lind E.; Wernersson L.-E. High-Performance Vertical III-V Nanowire MOSFETs on Si With g m > 3 MS/Mm. IEEE Electron Device Lett. 2020, 41, 1161–1164. 10.1109/LED.2020.3004716.

[ref15] Park M. H.; Kim H. J.; Kim Y. J.; Moon T.; Kim K.; Hwang C. S. Thin Hf x Zr 1- x O 2 Films: A New Lead-Free System for Electrostatic Supercapacitors with Large Energy Storage Density and Robust Thermal Stability. Adv. Energy Mater. 2014, 4, 1400610 10.1002/aenm.201400610.

[ref16] Park M. H.; Kim H. J.; Lee Y. H.; Kim Y. J.; Moon T.; Kim K.; Hyun S. D.; Hwang C. S. Two-Step Polarization Switching Mediated by a Nonpolar Intermediate Phase in Hf 0.4 Zr 0.6 O 2 Thin Films. Nanoscale 2016, 8, 13898–13907. 10.1039/C5NR08346J. 26726129

[ref17] Ushakov S. V.; Navrotsky A.; Yang Y.; Stemmer S.; Kukli K.; Ritala M.; Leskela M. A.; Fejes P.; Demkov A.; Wang C.; Nguyen B.-Y.; Triyoso D.; Tobin P. Crystallization in Hafnia- and Zirconia-Based Systems. Phys. Status Solidi B 2004, 241, 2268–2278. 10.1002/pssb.200404935.

[ref18] Zheng W.; Bowen K. H.; Li J.; Dabkowska I.; Gutowski M. Electronic Structure Differences in ZrO 2 vs HfO 2. J. Phys. Chem. A 2005, 109, 11521–11525. 10.1021/jp053593e. 16354043

[ref19] Lomenzo P. D.; Li S.; Pintilie L.; Istrate C. M.; Mikolajick T.; Schroeder U. Memory Window Enhancement in Antiferroelectric RAM by Hf Doping in ZrO_2_. IEEE Electron Device Lett. 2022, 43, 1447–1450. 10.1109/LED.2022.3189159.

[ref20] Pesic M.; Schroeder U.; Slesazeck S.; Mikolajick T. Comparative Study of Reliability of Ferroelectric and Anti-Ferroelectric Memories. IEEE Trans. Device Mater. Reliab. 2018, 18, 154–162. 10.1109/TDMR.2018.2829112.

[ref21] Lomenzo P. D.; Slesazeck S.; Mikolajick T.; Schroeder U. Thickness Scaling of AFE-RAM ZrO _2_ Capacitors with High Cycling Endurance and Low Process Temperature. In 2020 IEEE International Memory Workshop (IMW); IEEE, 2020; pp. 1–4. 10.1109/IMW48823.2020.9108146.

[ref22] Schenk T.; Yurchuk E.; Mueller S.; Schroeder U.; Starschich S.; Böttger U.; Mikolajick T. About the Deformation of Ferroelectric Hystereses. Appl Phys Rev 2014, 1, 041103 10.1063/1.4902396.

[ref23] Takagi S.; Toriumi A. Quantitative Understanding of Inversion-Layer Capacitance in Si MOSFET’s. IEEE Trans. Electron Devices 1995, 42, 2125–2130. 10.1109/16.477770.

[ref24] Nicollian E. H.; Brews J. R. MOS (Metal Oxide Semiconductor) Physics and Technology; Wiley*:* New York, 1982. 319–370.

[ref25] Persson A. E. O.; Athle R.; Svensson J.; Borg M.; Wernersson L.-E. A Method for Estimating Defects in Ferroelectric Thin Film MOSCAPs. Appl. Phys. Lett. 2020, 117, 242902 10.1063/5.0029210.

[ref26] Babadi A. S.; Lind E.; Wernersson L.-E. ZrO 2 and HfO 2 Dielectrics on (001) n-InAs with Atomic-Layer-Deposited in Situ Surface Treatment. Appl. Phys. Lett. 2016, 108, 132904 10.1063/1.4945430.

[ref27] Athle R.; Persson A. E. O.; Irish A.; Menon H.; Timm R.; Borg M. Effects of TiN Top Electrode Texturing on Ferroelectricity in Hf 1– x Zr x O 2. ACS Appl. Mater. Interfaces 2021, 13, 11089–11095. 10.1021/acsami.1c01734. 33625827 PMC8027987

[ref28] Robertson J. High Dielectric Constant Oxides. Eur. Phys. J.: Appl. Phys. 2004, 28, 265–291. 10.1051/epjap:2004206.

[ref29] Zacharaki C.; Tsipas P.; Chaitoglou S.; Evangelou E. K.; Istrate C. M.; Pintilie L.; Dimoulas A. Depletion Induced Depolarization Field in Hf 1–x Zr x O 2 Metal-Ferroelectric-Semiconductor Capacitors on Germanium. Appl. Phys. Lett. 2020, 116, 182904 10.1063/5.0007111.

[ref30] Pešić M.; Slesazeck S.; Schenk T.; Schroeder U.; Mikolajick T. Impact of Charge Trapping on the Ferroelectric Switching Behavior of Doped HfO _2_. Phys. Status Solidi A 2016, 213, 270–273. 10.1002/pssa.201532379.

[ref31] Mittmann T.; Szyjka T.; Alex H.; Istrate M. C.; Lomenzo P. D.; Baumgarten L.; Müller M.; Jones J. L.; Pintilie L.; Mikolajick T.; Schroeder U. Impact of Iridium Oxide Electrodes on the Ferroelectric Phase of Thin Hf _0.5_ Zr _0.5_ O _2_ Films. Phys. Status Solidi RRL 2021, 15, 2100012 10.1002/pssr.202100012.

[ref32] Athle R.; Blom T.; Irish A.; Persson A. E. O.; Wernersson L. E.; Timm R.; Borg M. Improved Endurance of Ferroelectric Hf _*x*_ Zr _1–*x*_ O _2_ Integrated on InAs Using Millisecond Annealing. Adv. Mater. Interfaces 2022, 9, 2201038 10.1002/admi.202201038.

[ref33] Liu H.; Wang C.; Han G.; Li J.; Peng Y.; Liu Y.; Wang X.; Zhong N.; Duan C.; Wang X.; Xu N.; Liu T.-J. K.; Hao Y. ZrO _2_ Ferroelectric FET for Non-Volatile Memory Application. IEEE Electron Device Lett. 2019, 40, 1419–1422. 10.1109/LED.2019.2930458.

[ref34] Persson A. E. O.; Athle R.; Littow P.; Persson K.-M.; Svensson J.; Borg M.; Wernersson L.-E. Reduced Annealing Temperature for Ferroelectric HZO on InAs with Enhanced Polarization. Appl. Phys. Lett. 2020, 116, 062902 10.1063/1.5141403.

[ref35] Lomenzo P. D.; Slesazeck S.; Hoffmann M.; Mikolajick T.; Schroeder U.; Max B.; Mikolajick T. Ferroelectric Hf 1-x Zr x O 2 Memories: Device Reliability and Depolarization Fields. In 2019 19th Non-Volatile Memory Technology Symposium (NVMTS); IEEE, 2019; pp. 1–8. 10.1109/NVMTS47818.2019.9043368.

[ref36] Schenk T.; Hoffmann M.; Ocker J.; Pešić M.; Mikolajick T.; Schroeder U. Complex Internal Bias Fields in Ferroelectric Hafnium Oxide. ACS Appl. Mater. Interfaces 2015, 7, 20224–20233. 10.1021/acsami.5b05773. 26308500

[ref37] Tagantsev A. K.; Stolichnov I.; Setter N.; Cross J. S. Nature of Nonlinear Imprint in Ferroelectric Films and Long-Term Prediction of Polarization Loss in Ferroelectric Memories. J. Appl. Phys. 2004, 96, 6616–6623. 10.1063/1.1805190.

[ref38] Zhou D.; Xu J.; Li Q.; Guan Y.; Cao F.; Dong X.; Müller J.; Schenk T.; Schröder U. Wake-up Effects in Si-Doped Hafnium Oxide Ferroelectric Thin Films. Appl. Phys. Lett. 2013, 103, 192904 10.1063/1.4829064.

[ref39] Kim H. J.; Park M. H.; Kim Y. J.; Lee Y. H.; Moon T.; Kim K. D.; Hyun S. D.; Hwang C. S. A Study on the Wake-up Effect of Ferroelectric Hf 0.5 Zr 0.5 O 2 Films by Pulse-Switching Measurement. Nanoscale 2016, 8, 1383–1389. 10.1039/C5NR05339K. 26511062

[ref40] Toprasertpong K.; Takenaka M.; Takagi S. Memory Window in Ferroelectric Field-Effect Transistors: Analytical Approach. IEEE Trans. Electron Devices 2022, 1–7119. 10.1109/TED.2022.3215667.

