# Supplementary material for: Ferroelectric-Antiferroelectric Transition of Hf1–xZrxO2 on Indium Arsenide with Enhanced Ferroelectric Characteristics for Hf0.2Zr0.8O2
Source: ACS Appl Electron Mater. 2022 Dec 14;4(12):6357–63. doi: 10.1021/acsaelm.2c01483 (PMC9798826; doi:10.1021/acsaelm.2c01483)
Supplement: Supplementary file 1 — el2c01483_si_001.pdf [file el2c01483_si_001.pdf]

Supporting Information

Ferroelectric-Antiferroelectric transition of $Hf_{1-x}Zr_xO_2$ on Indium Arsenide with enhanced ferroelectric characteristics for $Hf_{0.2}Zr_{0.8}O_2$

Hannes Dahlberg,[1, *] Anton E. O. Persson,[1] Robin Athle,[1, 2] and Lars-Erik Wernersson[1]

[1]*Electrical and Information Technology, Lund University, Box 118, 22 100, Lund, Sweden*

[2]*NanoLund, Lund University, Box 118, 22 100, Lund, Sweden*

Corresponding Authors*: hannes.dahlberg@eit.lth.se

The supporting information for this work includes the capacitance-voltage measurements that were used to determine the capacitive frequency dispersion (FIG S1), as well as a graphical representation of the average dispersion per frequency decade (FIG S2).

GIXRD measurements were performed and shown in FIG S3 for both MFM and MFS samples with 80% Zr.

Additionally, FIG S4 illustrates the MFIS response to the pulse train applied to MFS and MFIS in FIG 4. Here as well the decreasing pulse train results in a FE-like hysteresis for low biases (±2 V).

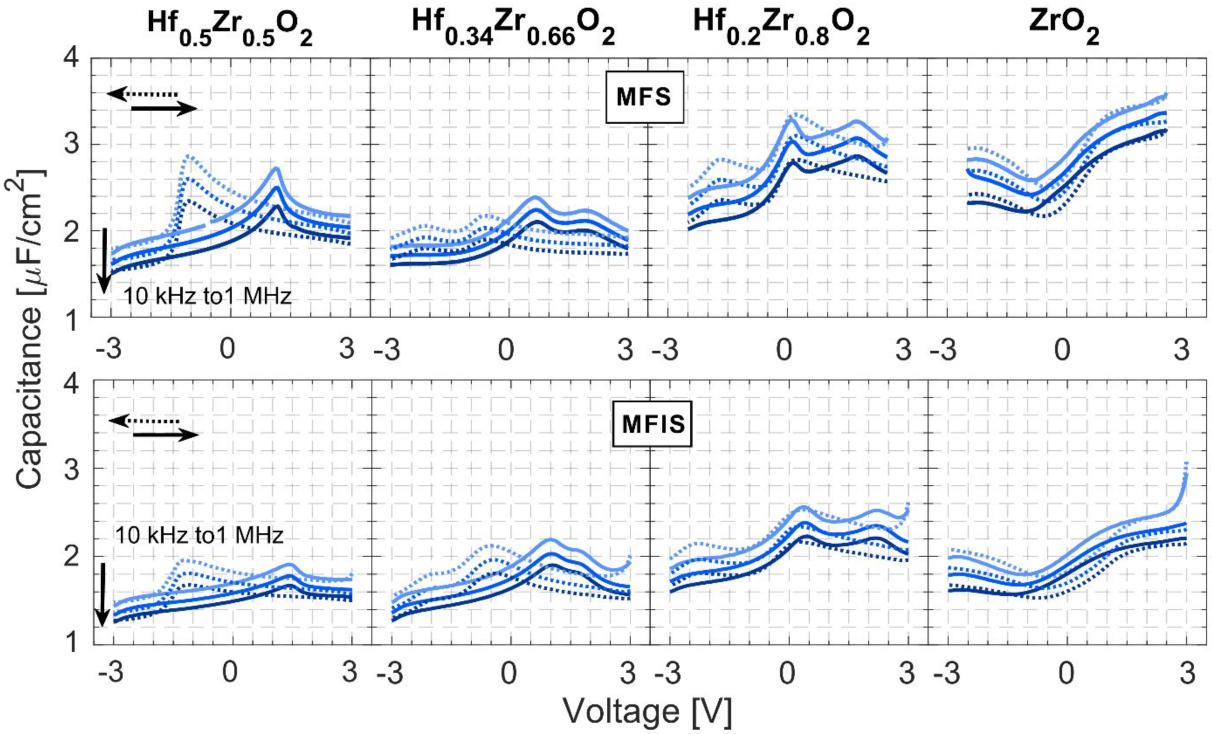

FIG. S1. Capacitance-voltage measurements for different AC bias frequencies (10 kHz to 1 MHz in direction of vertical arrow) for MFS and MFIS. Solid-line measurements (negative to positive bias sweep) are used for extracting the frequency dispersion.

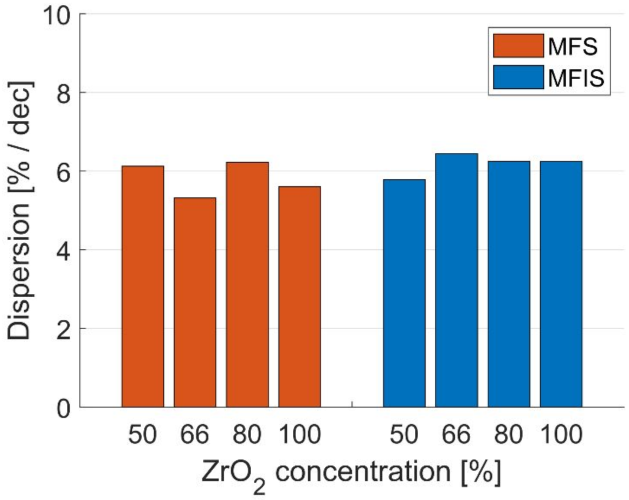

FIG. S2. Average frequency dispersion as capacitive change per AC frequency decade, determined from measured accumulation capacitance at 2 V (negative to positive bias sweep) for MFS and MFIS.

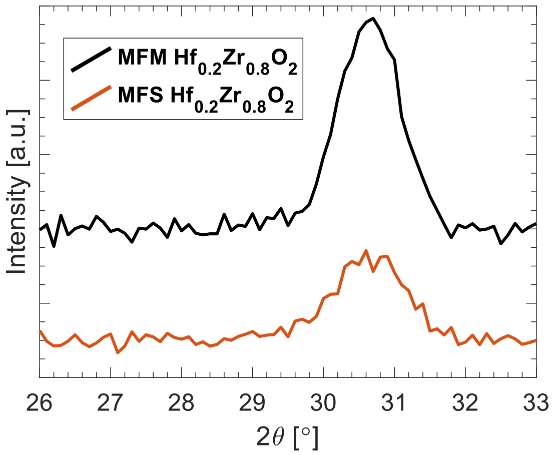

FIG. S3. GIXRD diffractogram of $Hf_{0.2}Zr_{0.8}O_2$ MFM and MFS devices at an incidence angle of 0.5°. An intensity peak at 30.7° for both samples indicates orthorhombic o(111)/tetragonal t(011) crystal phases [1].

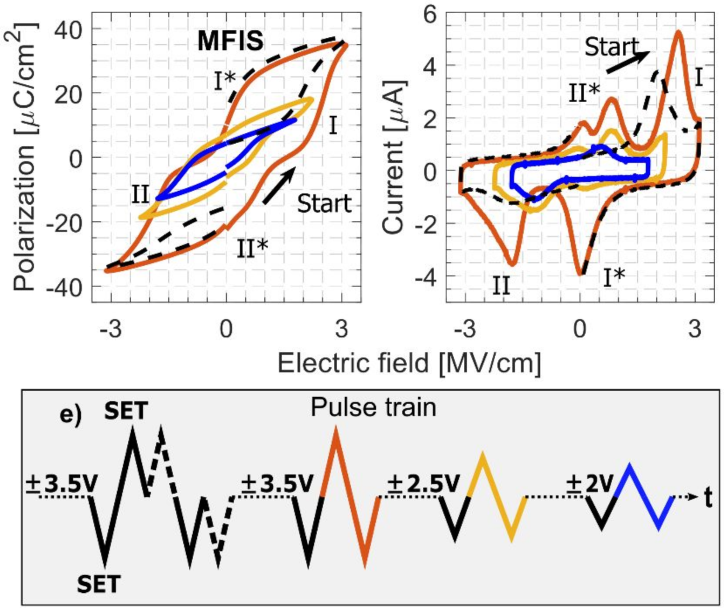

FIG. S4. Polarization-Voltage and Current-Voltage characteristics of MFIS $Hf_{0.2}Zr_{0.8}O_2$. The applied pulse train of varying amplitude and constant frequency is identical to that for MFS and MFM (FIG 4 (e)) but with voltage biases increased to ±3.5 V, ±2.5 V and ±2 V respectively.

References:

(1) Müller, J.; Böscke, T. S.; Schröder, U.; Mueller, S.; Bräuhaus, D.; Böttger, U.; Frey, L.; Mikolajick, T. Ferroelectricity in Simple Binary ZrO 2 and HfO 2. *Nano Lett* **2012**, *12* (8), 4318–4323. https://doi.org/10.1021/nl302049k.
